# The dynamics of financial performance and market performance in the context of Indian banking industry

**DOI:** 10.12688/f1000research.151628.2

**Published:** 2025-02-04

**Authors:** Ashok Sar, Kshirod Panigrahi

**Affiliations:** 1 School of Management, Kalinga Institute of Industrial Technology, Bhubaneswar, Odisha, 751024, India

**Keywords:** financial performance; stock market performance; capital adequacy ratio ; net non- performing asset ; CASA ratio; return on capital employed; net interest margin ; liquid asset to total asset; growth in share price

## Abstract

**Background:**

This study aims to gain insight into the effect of banks’ financial performance on their market performance. We conceptualized the research subject on the assumption that the financial performance of an organization is the most important criterion for triggering movement in its stock price. We explored various models and parameters to evaluate financial performance of banks and found CAMELS being one of the most comprehensive and appropriate model. We considered share price growth of banks to measure their stock market performance

**Methods:**

We collected financial and stock market data pertaining to 32 listed Indian banks for the period 2018 to 2022. The study has employed multiple linear regression analysis of panel data for evaluating the relationship between independent and dependent variables. We adopted panel regression for data analysis and used the Prais- Winsten regression with panel corrected standard errors, as the data suffers from contemporaneous cross-sectional correlation.

**Results:**

The results show that net non-performing assets, net interest margins, and return on capital have a significant negative impact on share price growth. The capital adequacy ratio and the current and savings account deposit ratios have a positive insignificant impact. The liquid asset-to-total asset ratio has a negative, insignificant impact. The coefficient of determination indicates that the share price growth of banks is more dependent on other factors which are not included in the regression analysis of this study.

**Conclusion:**

This study helps investors and bankers understand the limited impact of financial parameters on banks’stock prices and to look for other parameters which explain the stock price movement better.

## 1. Introduction

Despite the global issues of inflation and recession resurfacing, rallies in Indian banking stocks have continued. The banking sector is the foundation of the Indian economy as it channelizes the surplus in the form of investment to entrepreneurs in the form of credit. With the introduction of cutting-edge technology-driven business models, Indian banking has undergone transformation. Additionally, the government has been working to clean up the stressed balance sheets of public-sector banks. The “Make in India” initiative and the ongoing push for infrastructure investment are expected to increase credit demand. Banks will benefit substantially from this huge upsurge in credit-off takes. In 2022, the Nifty Bank index gained more than 11%, whereas the benchmark Nifty gained only 1.56%. Some of the most crucial measures for evaluating banking stock include net interest margins, credit-to-deposit ratios, capital adequacy ratios, return on equity ratios, and return on asset ratios. As the prospects of banks are heavily influenced by macro events and growth cycles, banking sector stocks can be highly volatile. One should invest in banking businesses if one is comfortable with the risks involved. Despite the fact that a slowdown in the global economy appears imminent, Indian banking stocks have been defying global trends. Accelerating credit demand and improving asset quality are anticipated to be favorable factors for these stocks. However, prior to making an investment, comprehensive research is required to avoid getting carried away by the excitement.

It has been a matter of great concern for investors to determine the most important factors influencing stock prices. Investors are engaged in analyzing the volume of data to detect predictable patterns of stock price movements. Ramakrishnan and Toppur (2016) state that stock prices are volatile, uncertain, complex, and ambiguous. Therefore, predicting the future performance of stocks is difficult and almost impossible. Anwaar (2016) maintains that stock prices are largely influenced by financial, monetary, and foreign trade policies, as well as other macroeconomic factors. He emphasized that investors make investment decisions mostly based on the financial information of a firm. Pradhan et al. (2014) find a causal relationship between economic, financial, and political risk factors and stock prices. Narayan et al. (2014) observed that industrial production, which was a surrogate for economic growth and the rate of exchange (currency depreciation), had a positive impact, whereas interest rate increase had an adverse impact on stock prices. Given the riskier nature of stock investments, it is imperative to understand the market dynamics and price determinants of a particular stock. Sharif et al. (2015) observed that internal factors, such as firm performance, management capability, corporate planning, and strategy, which are firm-specific, and external factors, such as macroeconomic parameters, government regulations, and market conditions, decide the price of a stock.

The price of a stock can be determined based on the current financial performance, consistency of past performance, and future revenue-generating potential of the firm. Consequently, the reaction of the market to financial performance should be linear across the firm, subject to other factors remaining constant. From this notion, we can reasonably assume that the financial performance of banks and their stock prices are strongly associated. We need to check the extent of the overall correlation and evaluate each financial performance dimension with respect to its impact on stock prices.

The Indian stock market is evolving rapidly, keeping pace with growing GDP. The market is maturing and offers new avenues of investment for both retail and institutional investors. Businesses find it easy to mobilize equity funding through the market. Against this backdrop, it is essential to understand the details of market performance and the dynamics thereof. This understanding will help investors make informed decisions about their portfolios. There has been more than 100 percent growth in the number of dematerialized accounts in just two years. The number was 40.9 million in March 2020, which has become 108.0 million in December 2022. As per the current market statistics, retail investors conduct 52% of daily transactions as against 29% and 19% by domestic and foreign institutional investors, respectively.

This study intends to assess the impact of the financial results of banks listed on the Indian stock exchange on the performance of their stock. The Indian banking industry has been chosen purposefully, as it is moving towards consolidation. Mergers of state-owned banks have resulted in a decrease in the number of public-sector banks and an increase in the size and business volume of anchor banks. As India is poised to register higher economic growth in the next ten years leveraging high domestic demand, Indian banks will play a significant role. Two Indian banks already appear on the list of the top 20 banks in terms of market capitalization. This number is expected to rise further as India is on the cusp of becoming 3 ^rd^ largest GDP by 2030, overtaking Japan and Germany, as per the S&P Global and Morgan Stanley report.

The second section covers previous studies and their contributions to the subject. The design, method, and approach are discussed in third section. Fourth section explains the result of the study. A thorough discussion and analysis of the findings are covered in the fifth part. The conclusions of this study are discussed in the final section.

## 2. Literature review

### 2.1 Financial performance

Sar (2017, 2018, 2019) states that a firm’s financial performance is assessed at the aggregate level using return on invested capital (ROIC), return on equity (ROE), the spread between ROE and the cost of equity, or the spread between ROIC and weighted average cost of capital (WACC). A firm is said to have superior financial performance if its ROE, ROIC, or economic rate of return compare well with a benchmark. Financial performance has a wider connotation, and can be evaluated using various parameters. Suhadak et al. (2019) suggest that financial performance is an indicator of the effectiveness and efficiency of an organization. Effectiveness indicates the capability of management to select the correct objective and tools to accomplish the objective. Efficiency refers to the optimum input utilization to produce the desired output. Previous studies have used several financial ratios, such as liquidity, leverage, activity, profitability, growth, and valuation, to evaluate financial performance.

The CAMELS rating system is an internationally acknowledged and accepted risk rating system employed by the central bank to evaluate the overall health of banks. Regulators and rating agencies use these to assess the strength of banks. Venkatesh and Suresh (2014) mentioned that the framework was first used in 1979 by the Federal Financial Institutions Examination Council (FFIEC). As stated by Banu and Vepa (2021) this model was recommended for measuring financial strength and weakness of banks by the Basel committee on banking supervision of the Bank of International Settlements’ (BIS) in 1988. In 1997, the model was expanded to include a sixth parameter, which is sensitive to market risk. In 1995, the Padmanabhan Committee recommended implementing the model in Indian Banks.

The model comprises six parameters: capital adequacy, asset quality, Management, Earnings, Liquidity, and Sensitivity". The strength of this model lies in its comprehensiveness in assessing capital sufficiency, operational efficiency, managerial capability, profit-generating ability, and financial stability. Unlike other ratings or regulatory ratios, the CAMELS rating is not made public. The regulatory authorities and top management of the respective banks use it only to understand and control potential risks. The Indian Reserve Bank of India (RBI), which is the central bank, uses a five-point rating system of five point scale, with one representing the highest rating and five representing the lowest.

Nguyen et al.(2020) narrating on the efficacy of the model articulated that the model was being adopted by three U.S. watchdogs. Nhan et al. (2021) emphasized the importance of this model as a barometer to check the overall performance and risk mitigation capability of banks. Describing the significance of the model, Handorf (2016) explained this as the most effective test to measure the soundness of a bank, and Dang (2011) considered this to be the most reliable tool for checking the health and safety of banks. The CAMELS model was chosen because it is the most acceptable and comprehensive framework for evaluating a bank’s financial performance and vulnerability to risk.

### 2.2 Market performance

Sar (2019) maintains that the market performance of a public limited company is assessed using measures associated with the performance of its shares in stock markets. Hobarth (2006) stated that market value versus book value and Tobin's ratio were measures used to evaluate a company's market performance. Market value to book value ratio indicates the market valuation of a company in comparison to its book value. Tobin’s Q is the ratio between the total market capitalization and the book value of an enterprise’s assets. He uses the following mathematical formula to measure the market performance of a company:

$$\Delta \;\text{Market value}\;t_{1}=\frac{\text{Market value}\;t_{1}-\text{Market value}\;t_{-1}}{\text{Market value}\;t_{-1}}\times 100$$

This formula calculates the share price growth of a company in percentage terms from *t*_-1_ to *t*_1_. This is the most common indicator used to evaluate market performance. The stock price of a firm is the true value, as the buyer of the stock paid the price for owning the share of that company. Though speculators manipulate stock prices in the short run, the price reflects the real value of the stock in the long run. The market performance of an enterprise is accurately reflected in its share price, as is the outsider’s view of the company. Unlike financial performance, market performance is an investor’s perspective on the company and industry. Stowe et al. (2010) mentioned two types of return from an investment in equity: capital gain, which is the amount of appreciation in the stock price, and dividends earned from equity during the ownership period of equity. Sharpe (1964) and Lintner (1965) propounded the capital asset pricing model (CAPM) based on the premise that stock prices were a function of the risk attached to it. The model explained that the expected yield from a stock or portfolio of stocks was the sum of the risk-free rate and risk premium attached to the risk of that stock or portfolio. The theory assumed that markets were efficient and investors were rewarded with a return equivalent to the quantum of risk. This model measured the systematic risk of a stock using its beta coefficient, which referred to the sensitivity of the return of a particular stock to the market portfolio. The CAPM model was challenged by Basu (1977), who found that CAPM failed to predict returns accurately for stocks with a high price earnings (PE) ratio because it underestimated the returns of those stocks. Banz (1981) also observed that the CAPM underestimated the average return of stocks with smaller market capitalization. Bhandari (1988) maintained that, for stocks, with a high debt-equity ratio, the actual return was higher than the return estimated based on the market beta. Rosenberg et al. (1985) and Stattman (1980) stated that the average yields of equity with high book-to-market ratios were higher than those indicated by their betas. Fama and French (1992) commented on the empirical failure of CAPM and observed that the estimation of the expected return forecast by market beta was supplemented by factors such as **size, PE ratio, debt equity and book to market ratio**.

### 2.3 Impact of financial performance on market performance

Sarjono and Suprapto (2020) in an attempt to assess the impact of CAMEL rating model on bank’s share price in Indonesian stock exchange found that movement of stock price was positively correlated with capital adequacy ratio (CAR), return on asset (ROA), return on equity (ROE) and net interest margin (NIM). In another study on the Indonesian stock exchange, Nugroho et al.(2020) found that, except for capital adequacy, the other four variables representing CAMELS had a negligible impact on stock prices. Ikechukwu and Owualah (2022) observed that, while capital adequacy, asset quality, and earnings had no impact, management capability and liquidity had significant negative influences on share prices. While conducting an impact assessment study of the CAMELS model on the share prices of Jordanian banks, Awwad (2022) found that asset quality, sensitivity, and liquidity had positive and substantial impacts. However, capital adequacy, management ability, and earnings had no impact.

Riani et al. (2020) observed that net profit margin, price-earnings ratio, total assets turnover ratio, and return on equity had a positive and significant relationship with share price, whereas debt equity and the current ratio had an insignificant impact on stock prices. Sumantri (2020) found that stock prices were substantially impacted by ROE and ROA. Wuryani et al. (2021) discover that capital adequacy affected stock prices, whereas profitability and liquidity did not. Awalakki and Archanna (2021) analyzed the impact of 11 independent variables on stock returns and concluded that price to book value (PB) and ROE were two parameters which had positive and significant impact on performance of stock.

Sharif et al. (2015) assessed the impact of eight variables on share prices and found that all eight variables had a positive and significant relationship with dividend yield (DY), which had a negative relationship. Anwaar (2016) examined the relationship between five independent variables displaying a firm’s performance and one dependent variable, stock returns, and found that return on assets (ROA) and net profit margin (NPM) had significant and positive effects on stock performance.

Nureny (2019) found that the (CAR) had a significant impact on share prices. In a similar study on the Indonesian stock exchange, Rusdiyanto et al. (2019) found that CAR and non-performing loans (NPL) had a substantial impact on stock prices. Benyamin and Endri (2019) and Al-qudah (2020) found that share price was strongly and positively impacted by Return on Equity (ROE). Agustyawati and Rais (2023) found that Earning per share (EPS), Net profit margin (NPM), and current ratio had significant impact on stock prices whereas Debt equity ratio and price earnings ratio had insignificant impact on stock prices. Gea and Tobing (2022) observed that return on asset (ROA) and earnings per share (EPS) influenced stock prices positively.

Hatem (2017) considered share price growth an appropriate parameter to measure stock market returns. He observed that growth in share price (SPG) was positively and strongly impacted by return on equity (ROE). However, a similar relationship was not found between return on asset (ROA) and SPG. Jape and Pauldhas (2021) conducted a study to understand the impact of economic variables such as the G-sec coupon rate, FII funds flow, and exchange rate on stock price movement. Trần Nha Ghi (2015) and Fathony et al. (2020) sought to determine the impact of a company's financial success on its stock return. In a similar effort to evaluate how different financial ratios affect stock returns, Chabachib et al.(2020) used “growth in share price” to measure stock performance.

### 2.4 Control variable

Nielsen and Raswant (2018) noted that control variables helped us measure the impact of extraneous variables which could influence the relationship between predictor and outcome variables. Bernerth and Aguinis (2016) remarked that it enabled the researcher to eliminate the scope of any alternative explanation other than the hypothesized relationship between the independent and dependent variables. Curado et al. (2024) viewed that it restricts the role of any other variable which does not explain the causal relationship among variables. It improves the efficiency of the model in terms of estimation of values of coefficient by removing statistical noise.

Based on previous studies, we found size of the bank would be the most appropriate control variable which might impact stock price of banks. Elsas et al. (2010) observed that diversified income streams and size of the bank impacted valuation of the bank. Goddard et al. (2004) and Mercieca et al. (2007) discovered that there was a positive relationship between size and profitability of banks. Similarly Petria et al. (2015) noticed positive relationship between size and return on average asset of banks. Researchers have observed that large banks leverage on economies of scale, economies of scope and diversification to augment their valuation. There is also contrary view which suggests an adverse relationship between size of the bank and its market valuation. Avramidis et al. (2018) discerned an inverse relationship between these two. Sakawa et al. (2020) found that large banks were engaged in more risky activities and hence there valuation was low. The studies which have found negative relationship between the two attributed it to higher cost of operation and lower return on asset.

### 2.5 Research gap

A review of past studies sheds light on the relationship between return on stocks and financial performance of various industries, including banks, using two to three sets of dependent variables. Many of these studies have suggested expanding the work in future research by using a greater number of variables. For further research, Rusdiyanto et al. (2019) suggested including more variables to determine stock price movement. Awalakki and Archanna (2021) find it very difficult to rely on a few parameters or a fixed model to predict stock prices. The results vary and financial parameters do not appear to have a uniform impact on stock prices. Although studies have measured the financial strength of Indian banks using the CAMELS parameters, the model has not been used to measure the impact on stock prices in the Indian banking industry. Many of these studies have suggested extending the use of the CAMELS model to assess market performance dynamics. Although the model has been extensively used to evaluate the overall performance of banks, it has not been used to assess the stock market performance of Indian banks.

Based on result of past studies, we formulated the following hypothesis:

H _0_
- There is no relationship between financial parameters consisting of CAMELS model and performance of stocks of Indian banks.

## 3. Methods

### 3.1 Sample and context

Despite global macroeconomic issues such as rising inflation, rising interest rates, and sluggish growth, the Indian banking industry is showing all healthy symptoms, such as adequate capital, rising profit, and steep fall in nonperforming assets. There are 12 state-owned banks, 22 private sector banks, 46 foreign banks, 12 small finance banks, four payment banks, and 43 regional rural banks. The total banking sector deposit is INR 177.34 trillion and bank credit is INR 133.04 trillion as on December 2022.

The Bombay Stock Exchange (BSE) and the National Stock Exchange (NSE) are the two Indian stock exchanges from which we obtain our sample. While collecting data, we found that there are a total of 32 banks listed on these two stock exchanges for at least five years. Therefore, the sample size for the study was equal to that of the whole population. For five years, stock market data were not available for the other banks, and they were not considered for the study.

### 3.2 Measures

Six independent variables were selected based on the CAMEL model. The last parameter of the CAMELS model - sensitivity to risk–has not been considered for the analysis, as appropriate data were not readily available for this parameter. The remaining five CAMEL parameters are measured using six financial ratios. Stock performance is measured by growth in share prices. Detail structure of the Table 1.

**Table 1.** Constructs, measures and references.

Construct	Measures	Reference
**C**-Capital Adequacy	Capital Adequacy Ratio (CAR)	Al Zaidanin (2020), Rusdiyanto et al. (2019), Banu & Vepa (2021), Sarjono & Suprapto (2020), Dang (2011), Nugroho et al. (2020), Maude et al. (2020), Sumantri (2020), Nureny (2019), Thisaranga & Ariyasena (2021), Wuryani et al. (2021)
**A**-Asset Quality	Net Non Performing Asset (NNPA)	Nureny (2019), Thisaranga & Ariyasena (2021) Banu & Vepa (2021), Sarjono & Suprapto (2020), Dang (2011), Nugroho et al. (2020), Nureny (2019)
**M**-Management efficiency	CASA Ratio (CASA)	Sarjono & Suprapto (2020), Indrajaya et al. (2022)
	Cost to Income ratio ( CTI)	Al Zaidanin (2020), Thisaranga & Ariyasena (2021)
	Return on Asset (ROA)	Anwaar (2016), Sarjono and Suprapto (2020), Maude et al. (2020), Nugroho et al. (2020), Sumantri (2020), Awalakki & Archanna (2021)
**E**-Earning ability	Return on Capital Employed (ROCE)	Banu & Vepa (2021), Das (2017), Hamidah (2015)
	Net Interest Margin (NIM)	Al Zaidanin (2020), Sumantri (2020), Sarjono & Suprapto (2020), Venkatesh & Suresh (2014), Dang (2011), Thisaranga & Ariyasena (2021), Nureny (2019)
	Return on Equity (ROE)	Anwaar (2016), Sarjono & Suprapto (2020), Muriithi (2018), Muriithi (2018), Sumantri (2020), Awalakki & Archanna (2021)
	Net profit Margin (NPM)	Anwaar (2016), Nugroho et al. (2020), Al Zaidanin (2020)
**L**-Liquidity	Liquid Asset to Total Asset (LATA)	Awwad (2022), Maude et al. (2020), Thisaranga & Ariyasena (2021), Trivedi (2013), Al Zaidanin (2020), Sathyamoorthi et al. (2017)
Performance of stock	Growth in share price (SPG)	Chabachib et al. (2020), Khan & Naz (2013), Hatem (2017), Anwaar (2016), Trần Nha Ghi (2015)
Size	Asset Volume (Lnta)	Elsas et al. (2010), Goddard et al. (2004), Petria et al. (2015), Sakawa et al. (2020)

Brief description of each variable is mentioned below.

**Capital Adequacy Ratio:** This ratio computes overall capital as a proportion of the bank's total risk-weighted assets. A higher ratio indicates better health. Capital acts as a shock absorber for banks during a liquidity crisis.

**Net NPA:** NPA stands for Non-performing assets, which are any asset that does not generate income for a stipulated period, as per the guidelines issued by the RBI. We obtain the Net NPA by deducting provisions from Gross NPA. Gross NPA consists of substandard, doubtful, and lost assets. A lower net NPA value is better for banks. The net NPA is always shown as a percentage of the total asset portfolio to understand the quality of a bank’s asset portfolio.

**CASA Ratio:** The ratio of demand deposit (current and savings) to the total deposit (demand plus term deposit) of a bank. A higher CASA ratio is desirable because demand deposits are less costly than time deposits. Therefore, a bank with a higher CASA ratio has a lower funding cost and a higher interest margin. The bank must provide a customized solution to capture the flow of funds and augment its service quality and product range to mobilize high-demand deposits. This reflects the efficiency of management.

**Return on Capital Employed:** This ratio signifies the earning capacity of a firm. Total earnings before interest and taxes are expressed as a percentage of the total capital employed. Total capital is computed by deducting current liabilities from total assets. A higher ROCE ratio indicates that companies efficiently utilize capital to obtain higher return.

**Net Interest Margin:** It calculated by deducting interest expenses from a bank’s interest income. It is computed by dividing the gap between investment returns and interest expenses by average earnings assets. This ultimately determines a bank’s profitability.

**Liquid Asset to Total Asset:** This ratio of current assets to total assets. The current asset indicates a bank’s liquidity strength. A higher liquidity ratio is considered to be healthier for banks.

**Growth in share price:** This measures the percentage growth in share price over the last year’s price. Growth was calculated based on the closing price of the stock.

**Size of the bank:** We have taken the asset size of the bank to measure the size of the bank. The natural logarithm of the asset volume has been used for estimation.

### 3.3 Data collection

The data were collected from 32 Indian banks listed on either of the two Indian stock exchanges.

NSE and BSE for 5 year time period from 2018 to 2022. Since we need six years of data to calculating 5 years share price growth, the closing share price of each bank from 2017 to 2022 was collected for the computation of stock price growth. Information was collected from the following websites: a list of 32 banks is given in Table 2.

**Table 2.** List of banks.

SL No	Name of Bank	Types of Bank
1	State Bank of India	Public Sector Bank
2	Punjab National bank	
3	Canara Bank	
4	Bank of Boroda	
5	Bank of India	
6	Indian Bank	
7	Union Bank of India	
8	Central Bank of India	
9	Indian Overseas Bank	
10	Punjab & Sind Bank	
11	Bank of Maharastra	
12	Uco bank	
13	HDFC Bank	Private Sector Bank
14	ICICI Bank	
15	Axis bank	
16	Kotak Mahindra Bank	
17	Indisind Bank	
18	Yes Bank	
19	RBL Bank	
20	City Union Bank	
21	DCB Bank	
22	Dhanalaxmi Bank	
23	Federal Bank	
24	IDBI Bank	
25	J & K Bank	
26	Karur Vyasya Bank	
27	Bandhan Bank	
28	South Indian Bank	
29	CSB Bank	
30	Karnatak Bank	
31	IDFC Bank	
32	Ujjivan Small Finance Bank Limited	Small Finance Bank

https://www.moneycontrol.com/; https://economictimes.indiatimes.com/; /https:/www.nseindia.com/; https://www.bseindia.com/.

### 3.4 Data analysis

Keeping in view the nature of the data, a panel regression model was chosen to evaluate the effect of Indian Banks’ financial results on stock market performance. This study adopted a multiple linear regression analysis of panel data to measure the relationship between the explanatory and predictor variables. The regression equation is constructed using share price growth as the dependent variable and the six CAMEL parameters as independent variables.

$${\mathrm{spg}}_{\mathrm{it}}=\alpha +\beta_{1}{\left(\mathrm{car}\right)}_{\mathrm{it}}+\beta_{2}{\left(\text{nnpa}\right)}_{\mathrm{it}}+\beta_{3}{\left(\text{casa}\right)}_{\mathrm{it}}+\beta_{4}{\left(\text{roce}\right)}_{\mathrm{it}}+\beta_{5}{\left(\mathrm{nim}\right)}_{\mathrm{it}}+\beta_{6}{\left(\text{lata}\right)}_{\mathrm{it}}+{Ɛ}_{\mathrm{it}}$$

(i = 1,2,3 … . 32 and t = 2018, 2019, … . 2022)

spg _it_ refers to the share price growth of bank i for period t. where Î ± is the intercept of the regression model. β _1_ to β _6_ are the slope coefficient for the independent variables and Ɛ _it_ is the error term.

## 4. Results

The observable explanatory variables were carefully chosen to ensure that they did not suffer from multicollinearity. It is known that multicollinearity adversely impacts the efficacy of the regression model, reduces the accuracy of estimation of the coefficient, and produces a distorted p-value that cannot be relied upon. Therefore, to determine the extent of correlation among the explanatory variables, a multicollinearity test using Variance Inflation Factors (VIF) was performed. Four variables with a VIF score of > 3 were eliminated, and six variables with VIF scores of < 3 and mean VIF score of 1.94. were considered for the regression analysis. The VIF details are given in Table 3.

**Table 3.** Variance Inflation Factors (VIF) result.

Variable	VIF	1/VIF
nim	2.92	0.341903
car	2.82	0.354705
roce	2.26	0.442378
nnpa	1.56	0.642735
casa	1.05	0.950788
lata	1.03	0.970061
Mean VIF	1.94	

We ensured that the data did not suffer from heteroscedasticity, contemporaneous cross-sectional correlation, or autocorrelation in the residuals, as the presence of these elements in the standard error could produce biased statistical inference. Cameron and Trivedi (2005) suggest that independent observations provide better information than correlated observations. Therefore, we cannot ignore the possible correlation between regression disturbances over time and between subjects. Bera et al. (2000) maintained that the standard error component model also addresses the serial correlation problem. Baltagi (2005) also confirmed that it addresses heteroscedasticity.

In our research, the data included time-series data. We must verify the degree of correlation between the values of the same variable over successive periods. This is known as an autocorrelation or serial correlation. The dataset exhibits first-order autocorrelation as per the Wooldridge test for autocorrelation. The associated details are presented in Table 4.

**Table 4.** Econometrics test results.

Test Description	Statistics	Probability
Wooldridge test for autocorrelation	11.478	0.0019
Modified Wald test for groupwise heteroscedasticity	1078.16	0.0000
Pesaran test of cross sectional dependence (FE)	11.742	0.0000
Pesaran test of cross sectional dependence (RE)	15.558	0.0000
Average correlation across units (FE)	0.532	
Average correlation across units (RE)	0.565	

Regression analysis assumes that the residuals or errors are homogeneous. The absence of this condition is known as heteroscedasticity. To ensure that the random error components are identically and independently distributed across the independent variables, we must verify the heteroscedasticity present in the collected data. The presence of heteroscedasticity in the panel data was validated using a modified Wald test for group-wise heteroscedasticity in a fixed-effect regression model, as suggested by Greene (2000).

Another important assumption of the panel data model is that the disturbances or errors available in the cross-section are independent. The cross-sectional dependence in the panel data is attributed to unobserved common elements in the error term. We used the CD test, as suggested by Pesaran (2004), to determine cross-sectional dependence. The test results demonstrate a high degree of dependence among cross-sectional data. Since the p-value is less than 0.001, we can reject the null hypothesis that there is weak cross-sectional dependence. If we assume that the unobserved common elements that are responsible for the cross-sectional dependence have no relationship with the independent variables, then the standard fixed-effects (FE) and random-effects (RE) models could be used, and the bias in the standard errors could be corrected by adopting the method of Driscoll and Kraay (1998). On the contrary, if these unobserved common elements that are responsible for the cross-sectional dependence are found to be correlated with the independent variables, then the FE and RE estimators will be unreliable or inconsistent and biased, and the approach proposed by Pesaran (2006) will be used as per De Hoyos and Sarafidis (2006). However, when the relationship between the two is not known, the choice of method is not clear.

Beck and Katz (1995) used Monte Carlo simulations to test the accuracy of panel corrected standard errors (PCSE) and to verify the efficiency of ordinary least squares (OLS) estimators. They were in favor of using OLS with PCSE instead of FE and RE models as well as the generalized least squares (GLS) estimator for panel data sets showing both heteroscedasticity and cross-sectional dependence.

Since all three problems (heteroscedasticity, contemporaneous cross-sectional correlation, and autocorrelation) are found in the error structure of our data, the ordinary least squares (OLS) with Therefore, PCSE was not appropriate for our study. Ardizzi et al. (2014) used the Prais–Winsten regression with Panel-Corrected Standard Errors (PCSE) when similar issues are observed in panel data. Therefore, we adopted the same model to adjust the standard errors appropriately. The associated details are presented in Table 5.

**Table 5.** Prais-Winsten regression, correlated panels corrected standard errors (PCSEs).

spg	Coef.	Std. Err.	Z statistics	P value	Lower 95%	Upper 95%
car	.0316538	.0192501	1.64	0.100	-.0060757	.0693833
nnpa	-.0499094	.0244486	-2.04	0.041	-.0978277	-.0019911
casa	.0108027	.0075503	1.43	0.152	-.0039957	.025601
roce	-.1076648	.041892	-2.57	0.010	-.1897716	-.025558
nim	-.0613966	.0270188	-2.27	0.023	-.1143524	-.0084408
lata	-.0469504	.1087801	-0.43	0.666	-.2601554	.1662546
_cons	-.216544	.5617477	-0.39	0.700	-1.317549	.8844613
Prob > chi ^2^	0.0209					
R-squared	0.2127					
Adj R-squared	0.1808					

The outcome demonstrates that the model is statistically significant because the p-value is less than 0.05. The regression model was a good fit for this study. Because the p-value of 0.0209 was < 0.05, the null hypothesis was rejected. This leads us to believe that there is a relationship between the CAMEL parameters and stock returns in Indian banks. The R ^2^ value signifies that 21.27% of the variance in share price growth is explained by six independent variables, whereas 78.73% of the variance is explained by variables not included in the regression model. The combined effect of all CAMEL parameters has a moderating effect on share price growth in the banking industry.

Control variables are effective tool to deal with endogeneity problem. Endogenity refers to a situation where the dependent variable is impacted by an omitted variable other than the independent variables thus leading to biased estimates. Hence we included size of the bank as a control variable in the regression model to evaluate the impact of this on the dependent variable.

There have been insignificant changes in the p values of all the variables after incorporation of the control variable. The R ^2^ value remained same which indicated that size of the bank did not have any impact on the valuation of the banks. The associated details are mentioned in Table 6.

**Table 6.** Prais-Winsten regression, correlated panels corrected standard errors (PCSEs) with control variable.

spg	Coef.	Std. Err.	Z statistics	P value	Lower 95%	Upper 95%
car	.0313204	.0191854	1.63	0.103	-.0062822	.0689231
nnpa	-.0497675	.0246428	-2.02	0.043	-.0980665	-.0014685
casa	.009912	.0062374	1.59	0.112	-.0023131	.0221372
roce	-.1121669	.0482897	-2.32	0.020	-.206813	-.0175207
nim	-.0522151	.0332186	-1.57	0.116	-.1173224	.0128922
lata	-.0402221	.1079057	-0.37	0.709	-.2517134	.1712692
Lnta	.0153738	.040015	0.38	0.701	-.0630542	.0938017
_cons	-.390036	.8522319	-0.46	0.647	-2.06038	1.280308
Prob > chi ^2^	0.0338					
R-squared	0.2127					
Adj R-squared	0.1752					

## 5. Discussion and further research

CAR has a regression coefficient of 0.0316 with a p-value of 0.10, which is > 0.05. This implies that for a 1% increase in the (CAR) the share price growth (SPG) will be impacted by 3.16%. Because the p-value is greater than 5%, the relationship is not significant. The NNPA has a regression coefficient of -0.0499 with a p-value of 0.04, which is < 0.05. This result indicates that a 1% increase in net Non-performing Asset (NNPA) causes a 4.99% change in share price growth (SPG). The negative sign indicates that when NNPA increases, the SPG is negatively impacted. The relationship between NNPA and SPG was significant, with a p-value > 0.05. The CASA has a positive impact on share price growth, but the relationship between the two is insignificant. ROCE, NIM, and LATA have a negative impact on SPG, and the relationship between ROCE and NIM with SPG is significant, as the p-value is < 0.05. The relationship between LATA and SPG was insignificant.

The findings of our analysis with respect to CAR are corroborated by the results of Sarjono and Suprapto (2020), Nugroho et al.(2020), Ikechukwu and Owualah (2022), and Nureny (2019). Our results regarding NNPA match those of Sarjono and Suprapto (2020) and Rusdiyanto et al. (2019). Ikechukwu and Owuala (2022) discovered a negative correlation between asset quality and share price. However, the relationship was found to be insignificant, whereas our results show that it is significant. The findings regarding LATA are supported by those of Anwaar (2016). Similar to our findings, Ikechukwu and Owualah (2022) also found a negative relationship between liquidity and share price, but unlike ours, their relationship is statistically significant. Our findings on managerial efficiency (CASA) and earning ability (ROCE and NIM) do not come from previous studies.

After incorporation of the control variable in the form of asset size of the bank we did not find any change in the R ^2^ value which suggested that size of the bank did not have any influence on the market performance of the banks. The findings resembled the conclusion of previous studies conducted by Avramidis et al. (2018), Sakawa et al. (2020) and Minton et al. (2019).

This study recommends further research on the subject to understand the key factors responsible for the performance of banking stocks. The study was not able to include sensitivity to market risk parameters in the CAMEL model. It also does not consider other important macroeconomic parameters, such as inflation and exchange rates, which are expected to impact stock returns. The impact of structural changes, such as the merger of public sector banks and COVID-19, was not included in the study. Stock market correlation and co-movement across geographies are also important factors that influence stock price movement, as illustrated by Evans and McMillan (2009) and Dos Santos and Lagoa (2017).

## 6. Implications for practice and research

The study would enable the senior management of the banks to evaluate the impact of various accounting performance parameters on the share price movement of banks and prioritize their focus accordingly. The results of the study unfolded that the stock markets closely looked at the non performing asset level and low cost deposit ratio which was explained by NNPA and CASA variables respectively. Higher level of good quality assets and lower cost of fund resulted in higher stock price. The moderate level of impact of the chosen CAMEL parameters will also prompt them to understand the significance of other factors such as macroeconomic indicators, geopolitical issues and performance of global stock markets. On the research respect the paper has made an attempt to measure the impact of financial performance by applying the CAMELS model on the stock market performance of the banks. The impact of asset quality on the market performance of bank has been reemphasized by the study.

## 7. Conclusion

Based on the results, the predictor variables selected for the study as per the CAMEL model have a moderate impact on the explanatory variable, that is, share price growth. This implies that there are other variables not considered in our study that are responsible for share price growth in the banking industry in India. As evident from the regression estimation, Net Non-performing asset (NNPA) measuring asset quality has a negative and significant impact on share price growth. This inference is statistically proven and commonsensical, as we know that any increase in nonperforming assets results in erosion of banks’ earnings. The net interest margin (NIM) and return on capital (ROCE) have negative and significant impacts. Surprisingly, these two variables, identified to measure earning ability, have a negative impact. This could be attributed to structural issues such as mergers in public sector banks and pandemics, which might have influenced stock prices. The impact of capital adequacy ratio (CAR) and CASA ratios is positive, but insignificant. A threshold Capital adequacy ratio being the regulatory requirement, and almost all banks maintain a reasonable level of CAR. Therefore, it might not be a powerful indicator of stock prices. The CASA ratio influences the cost of funds and the earnings of a bank. A higher CASA ratio entails lower interest expenses and lower cost of funds. However, the impact is positive but negligible. Liquid assets to total assets (LATA), which measures liquidity, has a negative and insignificant relationship with stock prices. The major takeaway is to make the research more comprehensive and include more valid explanatory variables to better predict stock prices.

## Ethics and consent

Ethical approval and written informed consent were not applicable.

## Data Availability

Figshare: Banking Industry Performance.xlsx, https://doi.org/10.6084/m9.figshare.25859644.v1 ( Sar, 2024) Data is available under the terms of CC By 4.0

## References

[ref71] Agustyawati D Rais M : The Effect Financial Performance on Stock Price (Case Study of Food Company Listed on Indonesia Stock Exchange BEI). *Int. J. Manag. Prog.* 2023;5(1):37–52. 10.35326/ijmp.v5i1.4121

[ref1] Al-qudah HA : The Impact of Financial ePrformance of Stock Prices of Jordanian Islamic Banks (During Period from 2010 to 2018). *Int. J. Econ. Financ. Issues.* 2020;10(1):228–234. 10.32479/ijefi.9157

[ref2] Al Zaidanin J : A Study on Financial Performance of the Jordanian Commercial Banks using the CAMEL Model and Panel Data Approach. *Int. J. Financ. Bank. Stud.* 2020;9(4):111–130. 10.20525/ijfbs.v9i4.978

[ref3] Anwaar M : Impact of Firms’ Performance on Stock Returns (Evidence from Listed Companies of FTSE-100 Index London, UK). *Global Journal of Management and Business Research:D Accounting and Auditing.* 2016;16(1):678–685. Reference Source

[ref4] Ardizzi G Petraglia C Piacenza M : Money launderung as a crime in the financial sector. *J. Money Credit Bank.* 2014;46(8):1555–1590. 10.1111/jmcb.12159

[ref72] Avramidis P Cabolis C Serfes K : Bank size and market value: The role of direct monitoring and delegation costs. *J. Bank. Finance.* 2018;93:127–138. 10.1016/j.jbankfin.2018.05.016

[ref5] Awalakki M Archanna HN : Impact of Financial Performance Ratios on Stock Returns – A Study With Reference to National Stock Exchange. *Int. J. Aquat. Sci.* 2021;12(03):2151–2167.

[ref6] Awwad MK : The Impact of CAMELS Model on Market Share Prices of Jordanian Commercial Banks, (Thesis), Middle East University (MEU). 2022; pp.6–49.

[ref7] Baltagi BH : *Econometric analysis of paneldata.* John wiley & Sons Ltd; Third ed. 2005; pp.11–104.

[ref8] Banu M Vepa S : A Financial Performance of Indian Banks Using CAMELS Rating System. *J. Contemp. Issues Bus. Govern.* 2021;27(1):2135–2153.

[ref9] Banz RW : The relationship between return and market value of common stocks. *J. Financ. Econ.* 1981;9(1):3–18. 10.1016/0304-405X(81)90018-0

[ref10] Basu S : Investment Performance of Common Stocks in Relation to their Price-Earnings Ratios: A Test of the Efficient Market Hypothesis. *J. Financ.* 1977;32(3):663–682. 10.1111/j.1540-6261.1977.tb01979.x

[ref11] Beck N Katz JN : What To Do (and Not to Do) with Time-Series Cross-Section Data. *Am. Polit. Sci. Rev.* 1995;89(3):634–647. 10.2307/2082979

[ref12] Benyamin IA Endri E : 'Determinants of Stock Returns of Building Construction Companies Listed on the Indonesia Stock Exchange Period 2012-2016. *Sch. J. Econ. Bus. Manag.* 2019;6(1):39–47. 10.21276/sjebm.2019.6.1.6

[ref13] Bera AK Escudero WS Yoon M : *Tests for the Error Component Model in the Presence of Local Misspecification.* Documento de Trabajo Nro., 22., Departamento de Economia, Universidad National de La Plata;2000; pp.7–20.

[ref73] Bernerth JB Aguinis H : A Critical Review and Best-Practice Recommendations for Control Variable Usage. *Pers. Psychol.* 2016;69(1):229–283. 10.1111/peps.12103

[ref14] Bhandari L : Debt/Equity Ratio and Expected Common Stock Returns: Empirical Evidence. *J. Financ.* 1988;43(2):507–528. 10.2307/2328473

[ref16] Cameron AC Trivedi P : *Microeconometrics: Methods and Applications.* Cambridge University Press;2005; pp.224–233. 10.1017/CBO9780511811241

[ref17] Chabachib M Setyaningrum I Hersugondo H : Does Financial Performance Matter ? Evidence on the Impact of Liquidity and Firm Size on Stock Return in Indonesia. *Int. J. Financ. Res.* 2020;11(4):546–555. 10.5430/ijfr.v11n4p546

[ref74] Curado C Oliveira M Schniederjans DG : Control variable use and reporting in operations management: a systematic literature review and revisit. *Manag. Rev. Q.* 2024;74(3):1809–1839. 10.1007/s11301-023-00348-2

[ref18] Dang U : The CAMEL rating system in banking supervision, (Thesis), Arcada University of Applied Sciences. 2011; pp.16–27.

[ref19] Das P : Saudi Journal of Business and Management Studies Impact of Return on Capital Employed On Company Performance-An Introspection in India. *Saudi J. Bus. Manag. Stud.* 2017;2(9):848–853. 10.21276/sjbms.2017.2.9.5

[ref20] De Hoyos RE Sarafidis V : Testing for cross-sectional dependence in panel-data models. *Stata J.* 2006;6(4):482–496. 10.1177/1536867x0600600403

[ref21] Dos Santos LGG Lagoa S : Herding behaviour in a peripheral European stock market: The impact of the subprime and the European sovereign debt crises. *Int. J. Bank. Account. Finance* 2017;8(2):174–203. 10.1504/IJBAAF.2017.087074

[ref22] Driscoll JC Kraay AC : Consistent covariance matrix estimation with spatially dependent panel data. *Rev. Econ. Stat.* 1998;80(4):549–560. 10.1162/003465398557825

[ref75] Elsas R Hackethal A Holzhäuser M : The Anatomy of Bank Diversification. *J. Bank. Finance.* 2010;34(6):1274–1287. 10.1016/j.jbankfin.2009.11.024

[ref23] Evans T McMillan DG : Financial Co-Movement and Correlation: Evidence from 33 International Stock Market Indices. *Int. J. Bank. Account. Financ.* 2009;1(3):215–241. 10.1504/IJBAAF.2009.022711

[ref24] Fama EF French KR : The Cross Section of Expected Stock Returns. *J. Financ.* 1992;47(2):427–465. 10.1111/j.1540-6261.1992.tb04398

[ref25] Fathony M Khaq A Endri E : The Effect of Corporate Social Responsibility and Financial Performance on Stock Returns. *Int. J. Innov. Technol. Creat. Change.* 2020;13(1):240–252.

[ref76] Gea K Tobing VLC : The Effect of Financial Performance on Stock Prices in Manufacturing Companies Listed on the Indonesia Stock Exchange. *J. Econ.* 2022;11(03):2022. Reference Source

[ref77] Goddard J Molyneux P Wilson J : Dynamics of Growth and Profitability in Banking. *Journal of Money, Credit and Banking.* 2004;36(6):1069–1090. 10.1353/mcb.2005.0015

[ref26] Greene WH : Econometric analysis. *Contributions to Management Science.* 2000. 10.1007/3-7908-1599-3_5

[ref27] Hamidah : EVA, ROCE, ROE, and EPS as Method of Assessment of Financial Performance and Its Effect on Shareholders’ Wealth: Evidence From Bank Listed at Indonesian Stock Exchange in 2011–2013. *Int. J. Sci. Res. Publ.* 2015;5(2):1–7.

[ref28] Handorf WC : CAMEL to CAMELS: The risk of sensitivity. *J. Bank. Regul.* 2016;17(4):273–287. 10.1057/jbr.2015.24

[ref29] Hatem BS : How Can We Measure Stock Market Returns? An International Comparison. *Int. Bus. Res.* 2017;10(5):121–126. 10.5539/ibr.v10n5p121

[ref30] Hobarth LL : *Modeling the relationship between financial indicators and company performance, An empirical study for US-listed companies.* WU Vienna University of Economics and Business;2006;269–288. PhD Thesis. Reference Source

[ref31] Ikechukwu OH Owualah SI : Evaluating the Impact of Camel Variables on the Share Price of Banks in Nigeria Evaluating the Impact of Camel Variables on the Share Price of Banks in Nigeria. *Int. J. Acad. Res. Account. Finance Manag. Sci.* 2022;1(3):295–308. 10.6007/IJARAFMS

[ref32] Indrajaya D Astuti M Maulidizen A : The Effect of Third-Party Funds, Capital Adequacy Ratio, Casa Ratio, Bi Rate, And Inflation Towards The Distribution of Credit Banking in Indonesia. *Int. J. Econ. Dev. Res.* 2022;2(3):171–185. 10.37385/ijedr.v2i3.282

[ref33] Jape S Pauldhas M : A STUDY AND ANALYSIS OF SELECTED STOCKS OF BANKING SECTOR LISTED ON BSE/NSE INDIA DURING CRISIS. *Int. J. Manag.* 2021;12(2):929–937. 10.34218/IJM.12.2.2021.090

[ref34] Khan Q Naz A : The Impact of Capital Structure and Financial Performance on Stock Returns “A Case of Pakistan Textile Industry”. *Middle-East J. Sci. Res.* 2013;16(2):289–295. 10.5829/idosi.mejsr.2013.16.02.11553

[ref35] Lintner J : The valuation of risk assets and the selection of Risky investments in stock portfolios and capital budgets. *Rev. Econ. Stat.* 1965;47(1):13–37. 10.2307/1926735

[ref36] Maude FA Tijjani MS Muazu SM : Effect of CAMELS Financial Indicators on Profitability of Systemically Important Banks (SIBs) in Nigeria. *Sokoto J. Manag. Stud.* 2020;1–21. 10.2139/ssrn.3666896

[ref78] Mercieca S Schaeck K Wolfe S : Small European banks: Benefits from diversification? *J. Bank. Finance.* 2007;31(7):1975–1998. 10.1016/j.jbankfin.2007.01.004

[ref79] Minton BA Stulz RM Taboada AG : ARE THE LARGEST BANKS VALUED MORE HIGHLY? *Rev. Financ. Stud.* 2019;32(12):4604–4652. 10.1093/rfs/hhz036 Reference Source

[ref38] Muriithi LK : *The effect of financial performance on stock price volatility of commercial bank listed at Nairobi Stock Exchange.* University of Nairobi;2018; pp.22–34. Research project.

[ref39] Narayan PK Narayan S Singh H : The determinants of stock prices: New evidence from the Indian Banking Sector. *Emerg. Mark. Financ. Trade.* 2014;50(2):5–15. 10.2753/REE1540-496X500201

[ref41] Nguyen AH Nguyen HT Pham HT : Applying the CAMEL model to assess performance of commercial banks: empirical evidence from Vietnam. *Banks Bank Syst.* 2020;15(2):177–186. 10.21511/bbs.15(2).2020.16

[ref42] Nhan DT Kim-Hung P Anh DT Van : The saftey of banks in Vietnam using CAMEL. *Adv. Decis. Sci.* 2021;25(2):1–34. 10.47654/v25y2021i2p158-192

[ref80] Nielsen BB Raswant A : The selection, use, and reporting of control variables in international business research: A review and recommendations. *J. World Bus.* 2018;53(6):958–968. 10.1016/j.jwb.2018.05.003

[ref43] Nugroho M Halik A Arif D : Effect of CAMELS Ratio on Indonesia Banking Share Prices. *J. Asian Finance Econ. Bus.* 2020;7(11):101–106. 10.13106/jafeb.2020

[ref44] Nureny : Financial Performance and Share Prices of Banks of State-Owned Enterprises in Indonesia. *Jurnal Ilmiah Ilmu Administrasi Publik.* 2019;9(2):315–326. 10.26858/jiap.v9i2.12335

[ref45] Pesaran M : Estimation and Inference in Large Heterogeneous Panels With a Multifactor Error Structure, CESIFO working paper No. 1331. 2006; pp.6–16.

[ref46] Pesaran MH : 'General Diagnostic Tests for Cross Section Dependence in Panels', Discussion Paper No. 1240. 2004; pp.2–42.

[ref81] Petria N Capraru B Ihnatov I : Determinants of Banks’ Profitability: Evidence from EU 27 Banking Systems. *Procedia Econ. Financ.* 2015;20(September):518–524. 10.1016/s2212-5671(15)00104-5

[ref47] Pradhan RP Hall JH Filho F d SP : Risk ratings and stock prices: The causal nexus in BRICS countries Risk ratings and stock prices. *Int. J. Bank. Account. Financ.* 2014;5(4):435–439. 10.1504/IJBAAF.2014.067021

[ref48] Ramakrishnan PR Toppur B : 'A Study of Banking Stocks in India to Develop a Model for Prudent Investment. *Univers. J. Manag.* 2016;4(9):477–487. 10.13189/ujm.2016.040902

[ref49] Riani M Muda I Rini ES : The Analysis of the Influence of Financial Performance on Stock Prices with Earning Growth as a Moderating Variable in Infrastructure, Utility and Transportation Sector Companies Listed on the Indonesia Stock Exchange. *Int. J. Innov. Res. Sci. Res. Technol.* 2020;5(8):897–904.

[ref50] Rosenberg B Reid K Lanstein R : Persuasive evidence of market inefficiency. *J. Portf. Manag.* 1985;11(3):9–16. 10.3905/jpm.1985.409007

[ref51] Rusdiyanto Soetedjo S Susetyorini : 'The Effect of Capital Adequacy Ratio (CAR), Net Profit Margin (NPM), Return on Assets (ROA), Non-Performing Loans (NPL), and Loan-to-Deposit Ratio (LDR) to Stock Prices in Banking Companies on the Indonesia Stock Exchange. *Int. J. Sci. Res.* 2019;8(7):1499–1510.

[ref82] Sakawa H Watanabel N Sasaki H : Bank valuation and size: Evidence from Japan. *Pac.-Basin Financ. J.* 2020;63:101403. 10.1016/j.pacfin.2020.101403

[ref52] Sar AK : Competitive advantage and performance: an analysis of Indian downstream Oil and Gas Industry. *Acad. Account. Financ. Stud. J.* 2017;21(2):21–34.

[ref53] Sar AK : Competitive advantage and performance: an analysis of Indian FMCG industry. *Acad. Account. Financ. Stud. J.* 2018;22(1):1–8.

[ref54] Sar AK : Impact of Competitive Advantage and Risk on Market Performance: A Study of Top 20 Companies as per Market Capitalization. *Int. J. Fin.* 2019;13(4):16–28. 10.17010/ijf/2019/v13i4/143126

[ref56] Sarjono H Suprapto AT : CAMEL ratio analysis of banking sector share price in Indonesia stock exchange. *Pal Arch. J. Egypt.* 2020;17(7):2213–2222.

[ref57] Sathyamoorthi CR Mapharing M Ndzinge S : Performance Evaluation Of Listed Commercial Banks In Botswana: The Camel Model. *Arch. Bus. Res.* 2017;5(10):142–158. 10.14738/abr.510.3783

[ref58] Sharif T Purohit H Pillai R : Analysis of Factors Affecting Share Prices: The Case of Bahrain Stock Exchange. *Int. J. Econ. Financ.* 2015;7(3):207–216. 10.5539/ijef.v7n3p207

[ref59] Sar A : Banking Industry Performance.xlsx.Dataset. *figshare.* 2024. 10.6084/m9.figshare.25859644.v1

[ref60] Sharpe WF : Capital asset prices: A theory of market equilibrium under conditions of risk. *J. Financ.* 1964;19(3):425–442.

[ref61] Stattman D : Book Values and Stock Returns'. *The Chicago MBA: A Journal of Selected Papers.* 1980;4(1):25–45.

[ref62] Stowe JD Robinson TR Pinto JE : Equity Asset Valuation. 2010; pp.144–154. Reference Source

[ref63] Suhadak K Handayani SR Rahayu SM : Stock return and financial performance as moderation variable in influence of good corporate governance towards corporate value. *Asian J. Account. Res.* 2019;4(1):18–34. 10.1108/AJAR-07-2018-0021

[ref64] Sumantri MB : The influence of financial performances toward stock's price of state owned bank listed on the indonesia stock exchange. *Pal Arch. J. Egypt.* 2020;17(10):3046–3053.

[ref66] Thisaranga K Ariyasena D : 'Effect of CAMEL model on bank performance: with special reference to listed commercial banks in Sri Lanka', Conferenec paper. *International Conference on Business Research, University Of Moratuwa, Sri Lanka.* 2021; pp.188–213.

[ref67] Trần Nha Ghi: The impact of capital structure and financial performance on stock returns on the firms in Hose. *Int. J. Inf. Res. Rev.* 2015;2(6):734–737.

[ref68] Trivedi JC : Performance Analysis of Bankex Banks Through Camel Model. *Manag. Dyn.* 2013;13(2):1–13. 10.57198/2583-4932.1109

[ref69] Venkatesh DJ Suresh MC : Comparative performance evaluation of selected commercial banks in kingdom of Bahrain using CAMELS method'. *Risk Management & Analysis in Financial Institutions EJournal.* 2014;1–42. 10.2139/ssrn.2418144

[ref70] Wuryani E Handayani S Mariana : The Effect of Financial Performance and Bank Size on Banking Stock Prices. *Proc. Int. Jt. Conf. Art. Humanit.* 2021;618:975–979.

