# Supplementary material for: The dynamics of financial performance and market performance in the context of Indian banking industry
Source: F1000Res. 2025 Feb 4;13:657. Originally published 2024 Jun 18. [Version 2] doi: 10.12688/f1000research.151628.2 (PMC11814959; doi:10.12688/f1000research.151628.2)
Supplement: Peer review history

# Reviewer report: Reviewer response for version 2

**DOI:** 10.5256/f1000research.176961.r364502

**Published:** 2025-02-11

**Authors:** Rizky Yudaruddin

**Affiliations:** 1 Mulawarman University, Samarinda, Indonesia

I have reviewed the revised manuscript submitted by the author and confirm that all previous comments have been adequately addressed. I have no further comments, and the manuscript is ready for further processing.

Is the work clearly and accurately presented and does it cite the current literature?

Partly

If applicable, is the statistical analysis and its interpretation appropriate?

Partly

Are all the source data underlying the results available to ensure full reproducibility?

Partly

Is the study design appropriate and is the work technically sound?

Partly

Are the conclusions drawn adequately supported by the results?

Partly

Are sufficient details of methods and analysis provided to allow replication by others?

No

Reviewer Expertise:

finance and banking

I confirm that I have read this submission and believe that I have an appropriate level of expertise to confirm that it is of an acceptable scientific standard.

# Reviewer report: Reviewer response for version 1

**DOI:** 10.5256/f1000research.166288.r339596

**Published:** 2024-12-03

**Authors:** Cacik Rut Damayanti

**Affiliations:** 1 Brawijaya University, Malang, East Java, Indonesia

Introduction:

1. The paragraph should have references, especially when the authors describe data in the banking sector.
2. The introduction should clearly describe the research problem in India, whether India has an issue with financial and market performance, to justify the importance of conducting research in this country.
3. The research gaps and novelty of this research should be illustrated in the introduction, such as how the CAMELS model has been under utilized for market performance analysis in the Indian banking sector.
4. While the abstract mentions the CAMELS model as a tool for assessing financial performance, it needs to justify why this model is particularly suitable for the study or how it stands out compared to other approaches.
5. Avoid generalizations, such as "Indian banking stocks have defied global trends," without providing data or citations to support these claims.

Literature Review

1. The review needs a more precise thematic structure. Discussions on financial performance, market performance, and the CAMELS model are scattered, making it hard for readers to follow the logical progression. I suggest organizing the literature review into clearly defined themes, such as: (a) Financial performance metrics. (b) Market performance and stock price determinants. (c) Prior studies using the CAMELS model. (d) Studies specific to the Indian banking sector.
2. The author should discuss the CAMELS model and how its application in past studies has been explored in depth. The review mentions the model but needs to evaluate its application across various contexts or its limitations critically and provides a detailed analysis of how the CAMELS model has been used in different studies to assess financial performance and market behavior. Highlight its strengths, weaknesses, and gaps in its application, especially in the Indian context.
3. The literature review primarily summarizes prior studies without critically analyzing their methodologies, findings, or relevance to the current research.
4. The review relies heavily on generalized studies, with limited inclusion of studies specific to the Indian banking sector or stock market.

Methods

1. The chapter briefly mentions the data sources (e.g., NSE and BSE websites) but does not provide enough detail on verifying the data’s accuracy or completeness. There is also no mention of handling potential biases in the data. I suggest including a detailed discussion of data quality assurance, such as cross-checking data across multiple sources or handling missing or outlier data points.
2.  I recommend conducting robustness checks, such as using alternative models (e.g., fixed effects, random effects, or dynamic panel models) or testing the results on different time frames or subsets of data to assess the reliability of the findings.
3. While the CAMELS model is central to the study, the chapter needs to adequately justify why specific variables (e.g., NNPA, ROCE) were selected or how they align with the study objectives.
4. The chapter mentions 32 listed banks as the sample but needs to discuss their representativeness or provide details about sample characteristics (e.g., public vs. private banks, size, regional focus).

Result

1. While the statistical significance of variables (e.g., NNPA, ROCE, and NIM) is discussed, the meaning of these findings in the context of the Indian banking sector is minimally interpreted.
2. Variables such as CAR, CASA, and LATA are found to be statistically insignificant, but their implications are not adequately explored.
3. The results are not compared with findings from previous studies, making it hard to contextualize the significance of the current study.
4. The chapter reports significance levels (p-values) but does not discuss their thresholds, the potential for Type I or Type II errors (e.g., 0.05 or 0.10), and the likelihood of false positives or negatives.
5. The chapter focuses on statistical findings but needs to discuss their practical implications for stakeholders like investors, policymakers, or bankers.
6. Summarize the results in the context of the hypotheses (e.g., "H0 is rejected for NNPA and NIM but not for CAR").
7. The negative impact of ROCE and NIM is surprising but should be discussed in more detail. I suggest discussing unexpected findings thoroughly, including potential reasons and their implications for the banking sector.
8. The chapter does not acknowledge any limitations in the results, such as potential biases, omitted variables or model restrictions.

Is the work clearly and accurately presented and does it cite the current literature?

Partly

If applicable, is the statistical analysis and its interpretation appropriate?

Yes

Are all the source data underlying the results available to ensure full reproducibility?

Partly

Is the study design appropriate and is the work technically sound?

Partly

Are the conclusions drawn adequately supported by the results?

Partly

Are sufficient details of methods and analysis provided to allow replication by others?

Partly

Reviewer Expertise:

Corporate governance, accounting and finance, sustainability.

I confirm that I have read this submission and believe that I have an appropriate level of expertise to confirm that it is of an acceptable scientific standard, however I have significant reservations, as outlined above.

# Reviewer report: Reviewer response for version 1

**DOI:** 10.5256/f1000research.166288.r328301

**Published:** 2024-11-18

**Authors:** Rizky Yudaruddin

**Affiliations:** 1 Mulawarman University, Samarinda, Indonesia

Summary of the Article

 This study aims to understand the effect of banks' financial performance on their market performance, based on the assumption that financial performance is a key driver of stock price movement. Using the CAMELS model to assess financial performance, the study analyses financial and market data from 32 listed Indian banks over the period 2018 to 2022 through multiple linear regression on panel data.

 Strengths of the Article

 The strength of this article lies in the use of the CAMELS model to measure the impact of financial performance on stock price movements in the Indian banking industry.

 Weaknesses of the Article

 1. Important variables such as the health crisis (COVID-19) have been ignored by the author

 2. Endogeneity problem

 3. Using stock performance measurements with the percentage growth in share price over the last year's price, completely ignores the dynamics of stock price fluctuations.

 Constructive Suggestions for Improvement

 1. The research period 2018-2022, is the period before and during COVID-19. The author needs to involve control variables such as COVID-19.

 2. The author needs to explain the existence of Endogeneity problems and how to overcome them

 3. The author needs to use daily data

 4. The author needs to do a robustness check

 5. The author needs to prepare a clear statement about the contribution of this study

 Dharani, M. et al., 2023 (Ref 1)

Is the work clearly and accurately presented and does it cite the current literature?

Partly

If applicable, is the statistical analysis and its interpretation appropriate?

Partly

Are all the source data underlying the results available to ensure full reproducibility?

Partly

Is the study design appropriate and is the work technically sound?

Partly

Are the conclusions drawn adequately supported by the results?

Partly

Are sufficient details of methods and analysis provided to allow replication by others?

No

Reviewer Expertise:

finance and banking

I confirm that I have read this submission and believe that I have an appropriate level of expertise to state that I do not consider it to be of an acceptable scientific standard, for reasons outlined above.

## References

[1] : Covid-19 pandemic and stock returns in India. *Journal of Economics and Finance* .2023;47(1) : 10.1007/s12197-022-09586-8 251-266 10.1007/s12197-022-09586-8

# Response

**Published:** 2025-01-12

**Authors:** Ashok Sar

**Affiliations:** School of Management, Kalinga Institute of Industrial Technology, Bhubaneswar, Odisha, India

I have addressed the 5 constructive suggestions in the revised manuscript. Size of the bank as a Control variable has been incorporated. Endogeneity problem has been discussed. Daily data for independent variables is not available as the financial parameters are available in the balance sheet and profit and loss statement of banks. With respect to robustness check Multiculinearity test through Variance Inflation Factors (VIF) was done. CD test as suggested by MH Pesaran (2004) has been done to find out the cross sectional dependence. Contribution of this study has been mentioned in a separate section.

# Reviewer report: Reviewer response for version 1

**DOI:** 10.5256/f1000research.166288.r314161

**Published:** 2024-08-26

**Authors:** Baranidharan Subburayan

**Affiliations:** 1 Christ University, Bangalore, Karnataka, India

**Summary of the Article  - The dynamics of financial performance and market performance in the context of Indian banking industry**

 The article examines the intricate relationship between financial performance and market performance in the Indian banking industry. By analyzing various financial metrics such as profitability, liquidity, and capital adequacy, alongside market indicators like stock prices and market capitalization, the authors aim to understand how these factors interact and influence each other. The findings suggest that financial performance significantly impacts market performance, with implications for both investors and banking institutions.

**Strengths of the Article**

 Comprehensive Coverage 

 The article covers a wide range of financial metrics, providing a holistic view of the banking industry’s financial health and its impact on market performance. This comprehensive approach adds depth to the analysis.

 Novelty and Relevance 

 The study addresses a relevant and timely issue in the Indian banking sector, which is crucial given the ongoing changes and challenges in the industry. The focus on the dynamics between financial and market performance adds a fresh perspective to the existing literature.

 Methodological Rigor 

 The methodology is sound, with clear articulation of the data collection process, analytical techniques, and statistical methods used. The use of appropriate models to analyze the relationship between financial and market performance enhances the study’s credibility.

**Weaknesses of the Article**

 Literature Review

 One of the significant weaknesses of the article is the literature review, which relies on research up to 2021. While the literature review is thorough, it lacks the inclusion of more recent studies that could provide updated insights and strengthen the foundation of the research. The use of present tense in some parts of the literature review is inconsistent with academic writing norms, where past tense is typically used to discuss previous research. This inconsistency may detract from the article’s professionalism and clarity.

 Implications for Practice and Research 

 The article would benefit from a more explicit discussion of the implications of the findings for both practitioners in the banking industry and future researchers. Adding this section before the conclusion would help to contextualize the findings and provide actionable insights.

**Constructive Suggestions for Improvement**

 Update the Literature Review The authors should consider including more recent studies, particularly those published after 2021. This would make the research more relevant and ensure that it reflects the latest trends and developments in the field. Consistency in Tense Usage Revising the literature review to ensure consistent use of past tense when referring to previous studies will improve the article’s readability and adherence to academic standards.

 Implications Section Introducing a section that discusses the managerial and research implications of the findings would greatly enhance the article. This would provide practical insights for industry professionals and highlight areas where further research is needed.

 The article makes a valuable contribution to the understanding of how financial performance influences market performance in the Indian banking industry. The comprehensive analysis and novel focus on this dynamic relationship are commendable. However, the inclusion of more recent literature, consistent tense usage, and a discussion of the practical and research implications would significantly strengthen the paper. The decision to approve the article with reservations is appropriate, given the need for these improvements. The suggestions provided here are intended to help the authors refine their work and ensure that it meets the highest academic standards.

Is the work clearly and accurately presented and does it cite the current literature?

Partly

If applicable, is the statistical analysis and its interpretation appropriate?

Yes

Are all the source data underlying the results available to ensure full reproducibility?

Yes

Is the study design appropriate and is the work technically sound?

Yes

Are the conclusions drawn adequately supported by the results?

Yes

Are sufficient details of methods and analysis provided to allow replication by others?

Yes

Reviewer Expertise:

Dr. S. Baranidharan, specializes in financial analytics, stock market analysis, financial econometrics, and economics.  The practical applications of financial theories, including the use of advanced statistical models and econometric techniques to analyze market trends, manage financial risks, and optimize investment strategies. He is also deeply involved in the study of derivatives markets, hedging strategies, and the development of financial tools for decision-making in uncertain environments.

I confirm that I have read this submission and believe that I have an appropriate level of expertise to confirm that it is of an acceptable scientific standard, however I have significant reservations, as outlined above.

# Response

**Published:** 2025-01-12

**Authors:** Ashok Sar

**Affiliations:** School of Management, Kalinga Institute of Industrial Technology, Bhubaneswar, Odisha, India

I have addressed the three sets of suggestions in the revised manuscript.
